# Plasma Neurofilament Light Chain as a Complementary Biomarker for Alzheimer’s Disease and Mild Cognitive Impairment: A Systematic Review and Meta-Analysis

**DOI:** 10.3390/ijms27198574

**Published:** 2026-09-25

**Authors:** Ka Young Kim, Ki Young Shin, Keun-A Chang

**Affiliations:** 1 Department of Nursing, College of Nursing, Gachon University, Incheon 21936, Republic of Korea; kykim@gachon.ac.kr; 2 Neuroscience Research Institute, Gachon University, Incheon 21565, Republic of Korea; 3 Bio-MAX Institute, Seoul National University, Seoul 08826, Republic of Korea; 4 Department of Pharmacology, College of Medicine, Gachon University, Incheon 21999, Republic of Korea

**Keywords:** Alzheimer’s disease, neurofilament light chain, blood-based biomarkers, mild cognitive impairment, Aβ positron emission tomography, early diagnosis

## Abstract

Alzheimer’s disease (AD) is a neurodegenerative disorder characterized by cognitive decline and memory loss, ultimately leading to a loss of independence and reduced quality of life. As current treatments are most effective in the early stages, reliable blood-based biomarkers are required for early diagnosis, therapeutic monitoring, and disease staging. Plasma neurofilament light chain (NfL), a marker of neuroaxonal injury, is a candidate biomarker; its value relative to established amyloid- and tau-based biomarkers is unclear. A systematic review and meta-analysis comparing plasma NfL with Aβ42, Aβ40, the Aβ42/Aβ40 ratio, and p-tau217 across cognitively normal (control, Con), mild cognitive impairment (MCI), and AD groups was conducted. Fourteen studies that met specific criteria were included. Plasma NfL and p-tau217 increased across disease stages. Aβ42 and Aβ42/Aβ40 were lower in AD/MCI than in Con; Aβ40 was similar across groups. Heterogeneity (I^2^ < 99%) was observed across biomarkers. Plasma NfL reliably reflected neuroaxonal injury and disease severity from the earliest clinically detectable stage of AD. Plasma NfL may be most valuable when combined with p-tau217/Aβ-based assays to provide a more comprehensive, blood-based assessment of both AD-specific pathology and neurodegeneration. Further studies are needed to validate its prognostic utility and establish clinically applicable cut-off values.

## 1. Introduction

Alzheimer’s disease (AD), the most common cause of dementia, is characterized by extracellular amyloid-β (Aβ) plaques and intracellular neurofibrillary tangles composed of hyperphosphorylated tau [1,2]. With the rapid development of disease-modifying therapies, the early identification of AD pathology and continuous monitoring during treatment have become increasingly pivotal [2,3]. Accessible and repeatable biomarkers are essential for guiding treatment initiation, monitoring therapeutic responses and disease progression, and supporting clinical decision-making [3,4]. However, amyloid positron emission tomography (PET) is expensive and has limited accessibility, whereas cerebrospinal fluid (CSF) biomarker testing is invasive and unsuitable for repeated assessment [3,5]. Therefore, reliable blood-based biomarkers are required for early diagnosis, treatment selection, therapeutic monitoring, and long-term follow-up [3,4,5].

Recent advances in ultrasensitive immunoassays have accelerated the clinical implementation of blood-based AD [3,5]. Among these, plasma-phosphorylated tau at threonine 217 (p-tau217) has demonstrated excellent performance in detecting cerebral amyloid pathology and differentiating AD from other neurodegenerative disorders [6,7,8]. Building on these findings, the Lumipulse G pTau217/Aβ42 Plasma Ratio, which measures plasma p-tau217 and Aβ42, became the first FDA-cleared blood-based in vitro diagnostic test for AD in 2025 [9,10]. This assay estimates the likelihood of cerebral amyloid pathology in patients with cognitive impairment and demonstrates high concordance with amyloid PET and CSF biomarkers, representing an important milestone in the clinical application of blood-based AD diagnostics [9,10,11].

Despite these advances, the blood-based diagnosis of AD continues to face limitations. AD is a heterogeneous disorder involving not only amyloid and tau pathology but also neuroaxonal injury, neuroinflammation, and progressive neurodegeneration [1]. Therefore, a single biomarker is unlikely to fully reflect the complex biological processes underlying a disease, highlighting the need for complementary biomarkers that capture the distinct pathophysiological mechanisms [1,3].

Neurofilament light chain (NfL) is a structural protein of myelinated axons that is released into the CSF and blood following neuroaxonal injury [5]. Blood NfL concentrations are elevated in patients with AD and mild cognitive impairment (MCI) and are associated with cognitive decline, brain atrophy, and disease progression [12]. These findings indicate that NfL reflects neuroaxonal injury and neurodegeneration rather than amyloid- or tau-specific pathologies. Therefore, NfL has emerged as a complementary biomarker that provides biological information beyond that provided by amyloid- and tau-based biomarkers. However, NfL is also elevated in several other neurodegenerative disorders, resulting in limited disease specificity and inconsistent diagnostic performance across studies [13,14].

Therefore, we conducted a systematic review and meta-analysis to evaluate the diagnostic utility of plasma NfL levels in patients with AD. Although previous studies have reported increased plasma NfL concentrations in patients with AD, their diagnostic performance varies substantially because of differences in study populations, analytical platforms, and diagnostic criteria. Additionally, the ability of plasma NfL levels to identify patients with MCI, an early clinical stage of AD, has not yet been systematically established. Consequently, the overall clinical utility of plasma NfL levels remains unclear. In this study, diagnostic performance of plasma NfL was quantitatively assessed in distinguishing patients with AD and MCI from cognitively normal individuals. Plasma NfL was compared with established blood biomarkers, including Aβ42, Aβ40, the Aβ42/Aβ40 ratio, and p-tau217, within the same meta-analytic framework, to determine its value as a complementary biomarker for both early diagnosis and disease evaluation.

## 2. Materials and Methods

### 2.1. Search Strategy

This systematic review adhered to the PRISMA 2020 guidelines [15]. A completed PRISMA-P checklist can be accessed from the Supplementary Materials, Supplementary S1. A systematic literature search was conducted across four electronic databases, PubMed, Embase, Web of Science, and the Cochrane Library, for records published until 10 January 2026. Search terms combined controlled vocabulary and free-text terms related to four concepts: AD and its prodromal stages (e.g., “Alzheimer disease” [Mesh], “Alzheimer*,” “Cognitive Dysfunction” [Mesh], “Mild Cognitive Impairment,” “MCI”), the plasma biomarker of interest (“Neurofilament Proteins” [Mesh], “neurofilament light chain,” “neurofilament light protein,” “NfL,” “NF-L”), blood-based sample types (“Blood” [Mesh], “Plasma” [Mesh], “Serum” [Mesh], “blood,” “plasma,” “serum”), and PET imaging (“Positron-Emission Tomography” [Mesh], “PET,” “PET scan,” “Amyloid PET,” “Tau PET,” “FDG-PET”). Boolean operators (AND/OR) were used to combine the four concept blocks. The retrieved records were imported to EndNote 25 (Clarivate, Philadelphia, PA, USA) for deduplication and screening. Following the electronic search, records were manually screened at the title, abstract, and full-text stages to identify studies that reported plasma NfL alongside plasma p-tau217 and/or blood Aβ measures (Aβ42, Aβ40, or the Aβ42/40 ratio) within the same cohort. This systematic review and meta-analysis was registered with the International Prospective Register of Systematic Reviews (registration number CRD420261426163) prior to data extraction and was conducted in accordance with the Preferred Reporting Items for Systematic Reviews and Meta-Analyses 2020 guidelines.

### 2.2. Study Selection

The study selection proceeded in three sequential stages: title screening, abstract screening, and full-text assessment. Two reviewers independently screened the records at each stage, and disagreements were resolved by discussion or adjudication by a third reviewer. The study identification and screening processes are summarized in the Preferred Reporting Items for Systematic Reviews and Meta-Analyses flow diagram (Figure 1).

Studies were eligible for inclusion if they met the following criteria: (a) the study population consisted of adults classified into the Con, MCI, or AD groups according to the diagnostic or grouping criteria defined in the original study; (b) plasma NfL was measured and reported together with at least one AD-related plasma biomarker (p-tau217 and/or amyloid-β measures, i.e., Aβ42, Aβ40, or Aβ42/40 ratio) in the same study population, and amyloid-PET data were available for the study; (c) group-level summary statistics (mean and standard deviation [SD], or median and interquartile range) and sample sizes were reported or could be obtained from the published article; and (d) the article was published in a peer-reviewed journal in English.

Studies were excluded if they were reviews, meta-analyses, conference abstracts, letters, commentaries, case reports, or animal/cellular studies; if the study population or topic was unrelated to AD or cognitive impairment; if the biomarker of interest was measured exclusively in the CSF or via imaging without a corresponding plasma measurement; if the study population, sample definition, or diagnostic grouping did not meet the predefined PICO criteria; or if the required outcome data (group-level biomarker summary statistics) were not reported and could not be obtained from the published article.

### 2.3. Data Extraction and Risk-of-Bias Assessment

Using a standardized extraction form, two reviewers independently extracted the following variables from each included study: first author, publication year, country, cohort/study name, diagnostic group definitions and their sample sizes, mean or median age, sex distribution, cognitive assessment instruments and scores, amyloid-PET tracer and positivity criteria (where applicable), assay platform used for biomarker quantification, and group-level summary statistics for plasma NfL, p-tau217, Aβ42, Aβ40, and Aβ42/40 ratio.

### 2.4. Effect Size Calculation and Statistical Synthesis

Meta-analyses were conducted using Review Manager 5.4 (RevMan 5; Cochrane Collaboration). Group-level outcome data for plasma Aβ42, Aβ40, Aβ42/40 ratio, p-tau217, and NfL were extracted as means with SDs where reported [16,17,18,19,20,21]. For the subset of studies that reported medians with interquartile ranges [22,23,24,25], values were transformed into estimated means and SDs using the method proposed by [26], which estimates the mean from the first quartile, median, and third quartile, and estimates the SD from the interquartile range and sample size under the assumption of an approximately normal underlying distribution. This approach has been shown through simulation studies to provide relatively unbiased estimates, particularly when *n* ≥ 25. The resulting estimated means and SDs were used for subsequent pooling in the same manner as the directly reported means and SDs. Pairwise MD with 95% CIs were calculated for each biomarker to quantify group differences between the AD and Con groups and between the MCI and Con groups. Positive values indicated higher biomarker concentrations in the AD or MCI group relative to the Con group. Pooled effect sizes were calculated separately for each biomarker (plasma Aβ42, Aβ40, Aβ42/40 ratio, p-tau217, and NfL). A random-effects model was applied a priori for all pooled comparisons, given the anticipated clinical and methodological heterogeneity across studies (e.g., differences in diagnostic criteria, assay platforms, and population characteristics). Inter-study heterogeneity was assessed using Cochran’s Q test and quantified using the I^2^ statistic.

## 3. Results

### 3.1. Study Selection and Characteristics

A total of 1349 records were identified using a database search (Embase 606, PubMed 265, Web of Science 414, Cochrane 64). After removing 466 duplicates, 883 records were subjected to title screening, of which 344 were excluded, leaving 539 for abstract screening. Following abstract review, 370 records were excluded and 169 full-text reports were assessed for eligibility; 155 were further excluded for not meeting patient/population, intervention, comparator/control, and outcome (PICO) criteria or lacking reportable outcome data, resulting in 14 studies being included in the quantitative synthesis (Figure 1).

The included studies were conducted in South Korea, China, Japan, Sweden, and Canada, and the participants were categorized into Control (Con), MCI, and AD groups. Diagnostic classification was based on cognitive assessment tools, such as Mini-Mental State Examination, Montreal Cognitive Assessment (basic) and Addenbrooke’s Cognitive Examination III (Chinese version) with amyloid-PET positivity or standardized uptake value ratio (SUVR)/centiloid values using various tracers (florbetapir, florbetaben, flutemetamol, AZD-4694). Amyloid-PET status was determined either by visual inspection according to validated regulatory reading criteria or by quantitative SUVR- or Centiloid-based classification, depending on the study. Plasma NfL, p-tau217, Aβ42, Aβ40, and the Aβ42/40 ratio were the five target biomarkers measured across all included studies (Table 1).

### 3.2. Quality Assessment

The risk of bias across the 14 studies was assessed using the Cochrane risk-of-bias tool (RevMan 5.4) (Figure 2). Considering the observational nature of most studies, some were rated as high-risk for randomization-related domains, whereas blinding, outcome assessment, and incomplete data domains were generally rated as low-risk. The “other bias” domain, by contrast, was judged unclear in approximately half of the studies, representing a comparatively weaker domain overall. Arslan et al. [27], Leuzy et al. [25], and Wang et al. [19] were rated as having low risk across all domains, whereas Brum et al. [22] and Kang et al. [29] showed high or unclear risk across multiple domains, indicating relatively greater methodological vulnerability.

### 3.3. Meta-Analysis of Plasma Biomarkers

Pooled mean differences (MD) were calculated using random-effects models for five plasma biomarkers: (A) Aβ42, (B) Aβ40, (C) the Aβ42/40 ratio, (D) p-tau217, and (E) NfL (Figure 3). Aβ42 was lower in patients with AD compared with controls (three studies, MD = −3.26, 95% confidence interval [CI] [−5.93, −0.59], I^2^ = 91%, *p* = 0.02) and was lower in patients with AD compared with patients with MCI (three studies, MD = −1.10, 95% CI [−1.66, −0.54], I^2^ = 0%, *p* = 0.0001). Aβ40 showed no marked difference across any of the three comparisons. The Aβ42/40 ratio was lower in patients with AD (eight studies, MD = −0.01, 95% CI [−0.02, −0.00], I^2^ = 97%, *p* = 0.002) and MCI (11 studies, MD = −0.01, 95% CI [−0.02, −0.01], I^2^ = 97%, *p* = 0.0006) compared to the controls.

Plasma p-tau217 was higher in patients with AD than in controls (eight studies, MD = 1.90, 95% CI [1.38, 2.42], I^2^ = 99%, *p* < 0.00001), in patients with MCI than in controls (11 studies, MD = 0.43, 95% CI [0.31, 0.54], I^2^ = 97%, *p* < 0.00001), and in patients with AD than with MCI (eight studies, MD = 1.02, 95% CI [0.65, 1.39], I^2^ = 97%, *p* < 0.00001). Similarly, NfL was higher in patients with AD than in the controls (eight studies, MD = 10.45, 95% CI [6.47, 14.43], I^2^ = 91%, *p* < 0.00001), in patients with MCI than in controls (11 studies, MD = 4.91, 95% CI [3.08, 6.74], I^2^ = 86%, *p* < 0.00001), and in patients with AD than with MCI (eight studies, MD = 5.33, 95% CI [2.79, 7.88], I^2^ = 75%, *p* < 0.0001).

## 4. Discussion

In this meta-analysis, five major blood biomarkers of AD, Aβ42, Aβ40, the Aβ42/Aβ40 ratio, p-tau217, and NfL, were compared across cognitively normal (Con), MCI, and AD groups. The patterns of change differed among the biomarkers across disease stages. Blood NfL levels were substantially higher in individuals with MCI than in the control, and were further increased in patients with AD. This consistent stepwise increase suggests that NfL may serve not only as a diagnostic biomarker for AD, but also as a marker of early cognitive impairment and disease progression.

Blood Aβ42 levels were substantially lower in patients with AD than in control individuals and patients with MCI, whereas no marked difference was observed between the Con and MCI groups. Aβ40 levels did not differ substantially among the three groups. For the Aβ42/Aβ40 ratio, no marked difference was observed between the AD and MCI groups; however, the ratio was substantially lower in both the AD and MCI groups than in the Con group. Variability in plasma Aβ concentrations has been reported, which may be influenced by analytical methods, preanalytical factors, and biological variability [21,32,33]. In particular, the Aβ42/Aβ40 ratio may be more robust than individual Aβ concentrations because it partially reduces variability unrelated to cerebral amyloid pathology [34,35]. Thus, the differences observed between Aβ42, Aβ40, and the Aβ42/Aβ40 ratio in the current analysis may reflect both biological changes across disease stages and methodological heterogeneity among studies.

Blood p-tau217 levels were substantially higher in patients with MCI and AD than in control individuals, and were substantially higher in the AD than in the MCI group. P-tau217 may therefore be altered during the early stages of cognitive impairment and increases with AD progression. This is consistent with previous studies showing that plasma p-tau217 is closely associated with cerebral amyloid and tau pathology and has a high diagnostic accuracy for AD [6,36]. Palmqvist et al. demonstrated that plasma p-tau217 could discriminate AD from other neurodegenerative disorders with high accuracy and was strongly associated with amyloid and tau pathology [6].

The clinical relevance of p-tau217 has been strengthened by the development of the Lumipulse G pTau217/Aβ42 plasma ratio. This assay combines plasma p-tau217 and Aβ42 to estimate the likelihood of cerebral amyloid pathology and became the first Food and Drug Administration-cleared blood-based in vitro diagnostic test for AD in 2025 [11,37,38]. In a clinical validation study supporting its use, the assay demonstrated high agreement with amyloid PET and cerebrospinal fluid reference standards [11,37]. These developments indicate that blood-based biomarkers targeting amyloid- and tau-related pathologies are rapidly transitioning from research settings to clinical practice.

However, AD cannot be fully characterized using amyloid and tau pathology alone. Neuroaxonal injury and progressive neurodegeneration are the major components of the disease process [12,39]. From this perspective, the current findings regarding NfL are particularly noteworthy. NfL is a structural component of the neuronal cytoskeleton that is released into the CSF and blood following neuroaxonal injury [12]. Unlike Aβ- and p-tau-based biomarkers, NfL primarily reflects the intensity of neuroaxonal damage rather than AD-specific amyloid or tau pathology [12,39].

In the present meta-analysis, blood NfL increased stepwise from the control to MCI and from MCI to AD, with statistical differences in all three pairwise comparisons. Elevated blood NfL levels in patients with MCI and AD and associations between higher NfL concentrations and cognitive decline, brain atrophy, and clinical progression have been reported [13,40]. Preische et al. demonstrated that longitudinal increases in serum NfL were associated with subsequent neurodegeneration and clinical progression, even during the pre-symptomatic phase of familial AD [13]. These findings support the hypothesis that blood NfL levels reflect the intensity and progression of neurodegenerative processes.

The marked difference between the control and MCI groups observed in the current meta-analysis supports the potential value of NfL in detecting early-stage neuroaxonal injury. Higher blood NfL concentrations in individuals with MCI than in cognitively normal controls have been reported, although the magnitude of these differences has varied across studies [40,41,42]. The current findings suggest that increased blood NfL levels are detectable before the development of clinically overt AD dementia. Furthermore, the markedly higher NfL levels in AD than in MCI indicate that NfL may provide information not only on early cognitive impairment, but also on the severity or rate of neurodegeneration.

The complementary relationship between phosphorylated tau and NfL is also supported by studies of p-tau species other than p-tau217. In particular, plasma p-tau181 and NfL have been shown to increase across the AD continuum and to be associated with cognitive decline and neurodegeneration, although they appear to reflect distinct aspects of AD pathophysiology [43,44,45]. Longitudinal studies have demonstrated that plasma p-tau181 and NfL show different trajectories during disease progression and provide complementary information regarding subsequent AD-related neurodegeneration and cognitive decline [43,44]. Whereas p-tau181 is more closely associated with AD-specific amyloid and tau pathology, NfL primarily reflects neuroaxonal injury and neurodegeneration and is therefore less specific to AD [43,44,45]. In addition, p-tau231 has emerged as a promising marker of early AD-related pathology; studies evaluating p-tau181, p-tau231, and NfL in parallel have shown stronger associations of p-tau181 and p-tau231 with cerebral amyloid pathology, whereas NfL provides complementary information related to neuronal injury and cognitive impairment [46]. Collectively, these observations suggest that the complementary relationship between phosphorylated tau and NfL may represent a broader biological relationship between AD-specific pathology and downstream neuroaxonal injury rather than a feature unique to p-tau217.

These characteristics suggest that NfL and p-tau217/Aβ-based biomarkers, including the Lumipulse assay, may have complementary rather than competing roles. The Lumipulse p-tau217/Aβ42 ratio is designed primarily to identify AD-related amyloid pathology [11,37], whereas NfL reflects neuroaxonal injury and neurodegeneration [12,39]. Thus, p-tau217- and Aβ-based biomarkers may provide information regarding the presence of AD-related pathology, whereas NfL offers insights into the extent of ongoing neuronal injury. Therefore, a combination of these biomarkers could provide a more comprehensive blood-based assessment of AD-specific pathology and downstream neurodegeneration.

Nevertheless, the lack of disease specificity of NfL should not be regarded solely as a limitation, as it may also have clinical value in reflecting neuroaxonal injury associated with coexisting pathologies. Mixed pathologies are common in older individuals with cognitive impairment, and cerebrovascular disease and Lewy body pathology may coexist with AD and contribute to cognitive decline and disease progression [47,48]. Because NfL reflects neuroaxonal injury irrespective of its underlying etiology, elevated blood NfL in an individual with evidence of AD-specific amyloid or tau pathology may reflect not only AD-related neurodegeneration but also the additional burden of coexisting vascular or neurodegenerative processes. Supporting this possibility, plasma NfL has been associated with vascular brain damage and vascular cognitive impairment [49]. Moreover, in patients with dementia with Lewy bodies (DLB), elevated plasma NfL has been associated with greater cognitive impairment and subsequent cognitive decline [50,51]. Recent evidence further indicates that plasma NfL in DLB is associated with MRI measures of cerebral atrophy as well as cerebrovascular lesion burden, including higher Fazekas scores [52].

These findings suggest that NfL may complement AD-specific biomarkers by providing information on the overall burden of neuroaxonal injury that is not captured by amyloid- or tau-specific biomarkers alone. Thus, in patients with positive AD-specific biomarkers, a disproportionately elevated NfL concentration could raise the possibility of additional neuronal injury related to co-pathologies and may prompt further clinical or imaging evaluation. However, NfL cannot identify the specific cause of neuroaxonal injury and therefore cannot by itself distinguish vascular injury, Lewy body disease, or other neurodegenerative disorders. NfL should consequently be interpreted in conjunction with clinical findings, disease-specific biomarkers, and neuroimaging when co-pathology is suspected. In addition, blood NfL concentrations are influenced by age and several medical conditions, including renal dysfunction, which should be considered when establishing clinically applicable reference ranges and cutoffs [45].

Collectively, the biomarkers evaluated in the current meta-analysis appear to reflect different aspects of AD pathophysiology. In particular, blood NfL showed a consistent stepwise increase across the control, MCI, and AD stages. This suggests that NfL may be valuable as a complementary blood biomarker for detecting early neuroaxonal injury and assessing disease progression, rather than as a stand-alone marker of AD-specific pathology. As p-tau217/Aβ-based assays such as Lumipulse become increasingly integrated into the clinical assessment of amyloid pathology, incorporating NfL may provide a more comprehensive blood-based approach that captures both AD-related pathology and the extent of neurodegeneration.

This study had several limitations. The analysis was restricted to data available from the included studies, which may have limited the generalizability of the results. Substantial heterogeneity remained in several pooled analyses, and the risk-of-bias assessment indicated that two studies with high or unclear risk across multiple domains, Brum et al. [22] and Kang et al. [29], contributed relatively large sample sizes to the pooled estimates for NfL and p-tau217; these methodological concerns, together with the use of different analytical platforms across cohorts, may have partly contributed to the observed heterogeneity (I^2^ up to 99%). In addition, the included cohorts used different amyloid-PET tracers and quantification approaches (visual read vs. SUVR/Centiloid); although amyloid-PET data were not a primary analytic variable, this may have contributed an additional source of between-study variability. Furthermore, because tau-PET or CSF/plasma tau-based AT staging was not used as a study selection criterion, formal A+T+ classification could not be consistently determined across the included cohorts, limiting interpretation of the observed plasma NfL differences, particularly within the heterogeneous MCI group. Although leave-one-out sensitivity analyses were conducted, pooled estimates were not formally stratified by risk of bias level; future meta-analyses with a larger number of eligible studies should consider risk-of-bias-based sensitivity or subgroup analyses. Longitudinal evidence was limited, restricting the ability to establish the prognostic value of NfL for progression from MCI to AD dementia. Moreover, as NfL reflects neuroaxonal injury rather than AD-specific pathology, its use as a stand-alone diagnostic marker is inherently limited [12,53,54].

Therefore, large prospective, longitudinal, and ethnically diverse cohorts are required to validate the diagnostic and prognostic performance of blood NfL. Blood NfL is unlikely to replace established CSF or imaging-based approaches. Instead, multimodal strategies that integrate NfL with amyloid- and tau-specific blood and CSF biomarkers, neuroimaging, and genetic information could offer greater diagnostic and prognostic accuracy [39]. The standardization of analytical methods and data reporting is essential to reduce inter-study variability. Future studies should determine whether adding NfL to p-tau217/Aβ-based diagnostic algorithms improves early detection, disease staging, and longitudinal monitoring of AD. Evaluating the cost-effectiveness and clinical utility of these integrated strategies is important for their translation into routine clinical practice.

## 5. Conclusions

Plasma NfL increased progressively across the Con–MCI–AD continuum, supporting its potential as a blood-based biomarker of early neuroaxonal injury and disease progression in AD. Although plasma NfL lacks disease specificity and cannot substitute for amyloid- or tau-specific biomarkers such as p-tau217, its consistent stepwise elevation, including the marked difference already detectable at the MCI stage, indicates that it can provide complementary information on the severity of ongoing neurodegeneration that pathology-specific markers alone do not capture. Integrating plasma NfL with p-tau217/Aβ-based assays, such as the recently FDA-cleared Lumipulse G pTau217/Aβ42 plasma ratio, may enable a more comprehensive blood-based framework that captures both AD-specific pathology and the extent of neurodegeneration.

Validation in large-scale, prospective, longitudinal, and ethnically diverse cohorts, together with the standardization of analytical platforms and reporting, will be essential before plasma NfL can be incorporated into routine clinical algorithms for the early diagnosis, staging, and monitoring of AD.

## Figures and Tables

**Figure 1:**
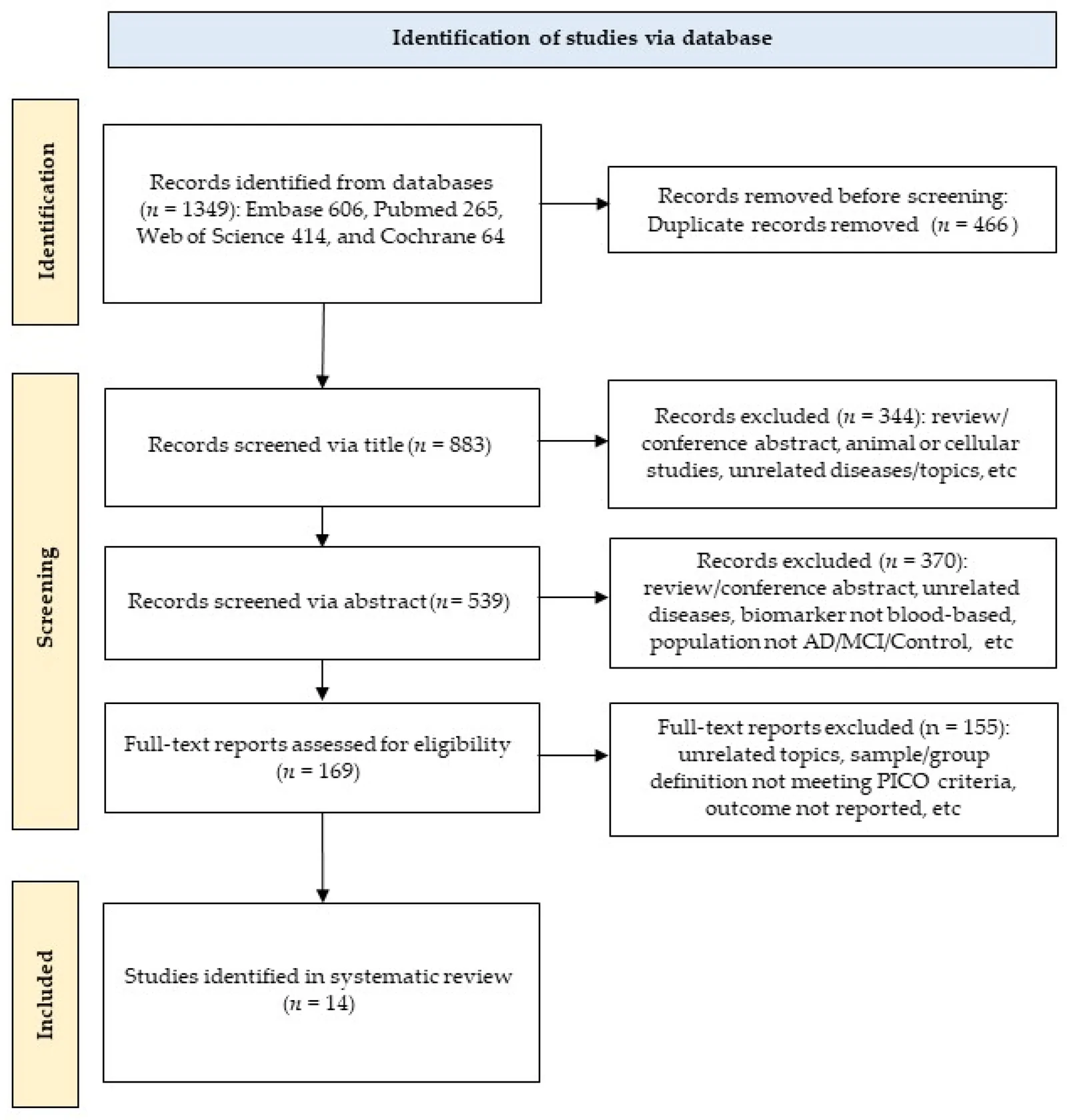
Flow chart.

**Figure 2:**
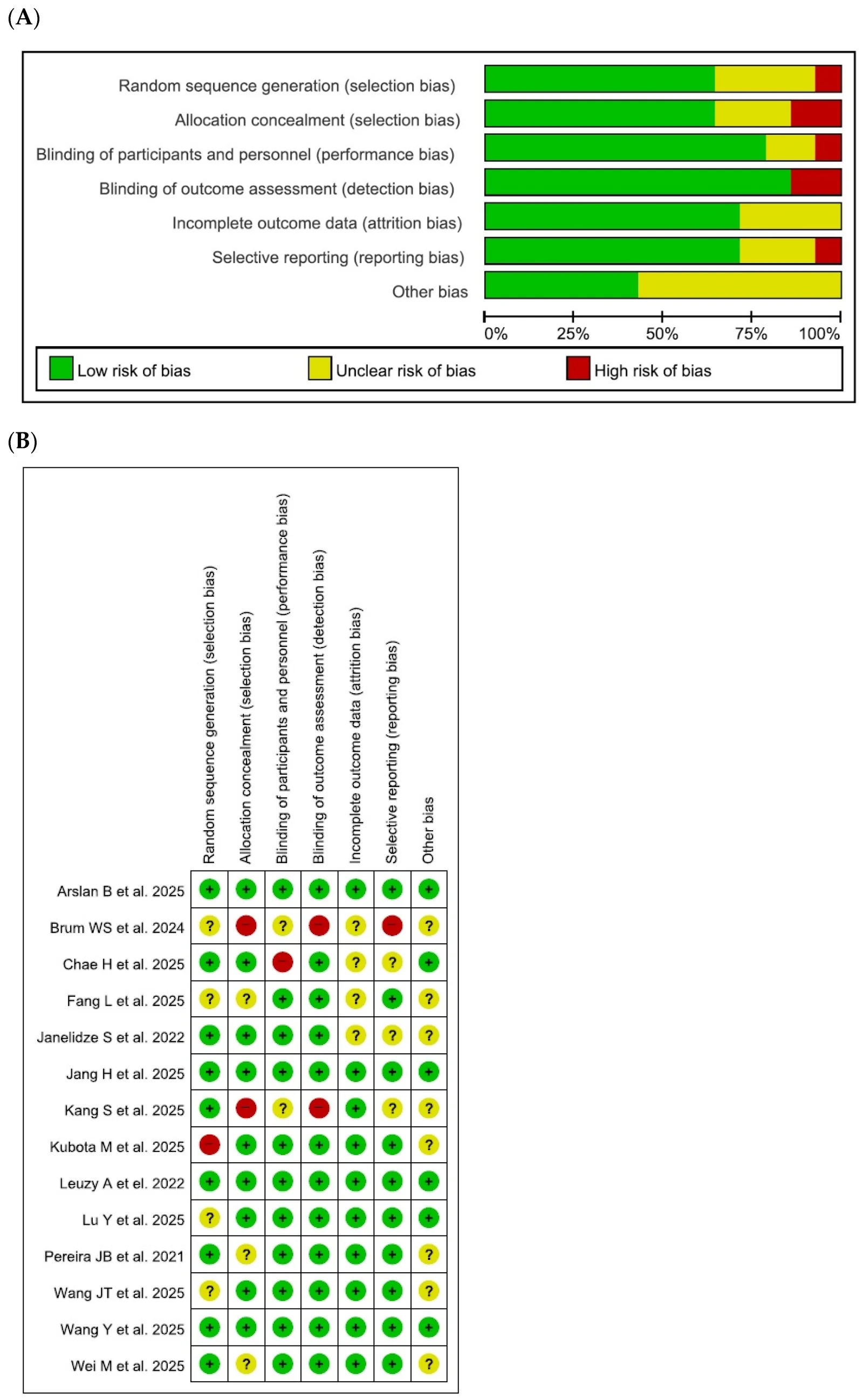
Quality assessment and bias risk assessment bar chart. Different colors (green, red, yellow) and symbols (“+”, “−”, “?”) represent “low risk of bias,” “high risk of bias,” and “unclear,” respectively. The quality assessment chart includes the percentage of bias risk at each level graph and the level of applicability concerns for each specific item in the study. (**A**) Risk of bias graph. (**B**) Risk of bias summary [16,17,18,19,20,22,23,24,25,27,28,29,30,31].

**Figure 3:**
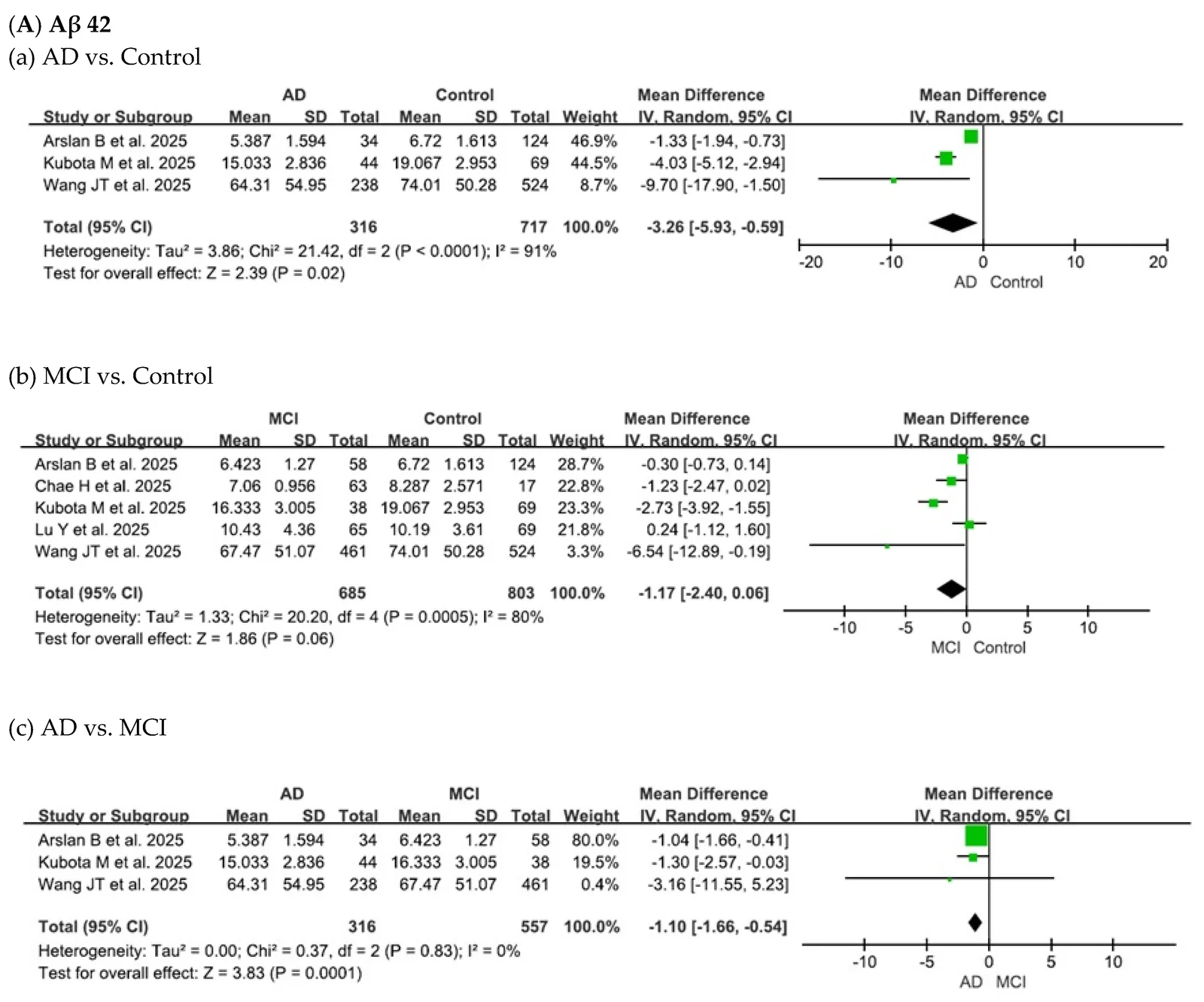

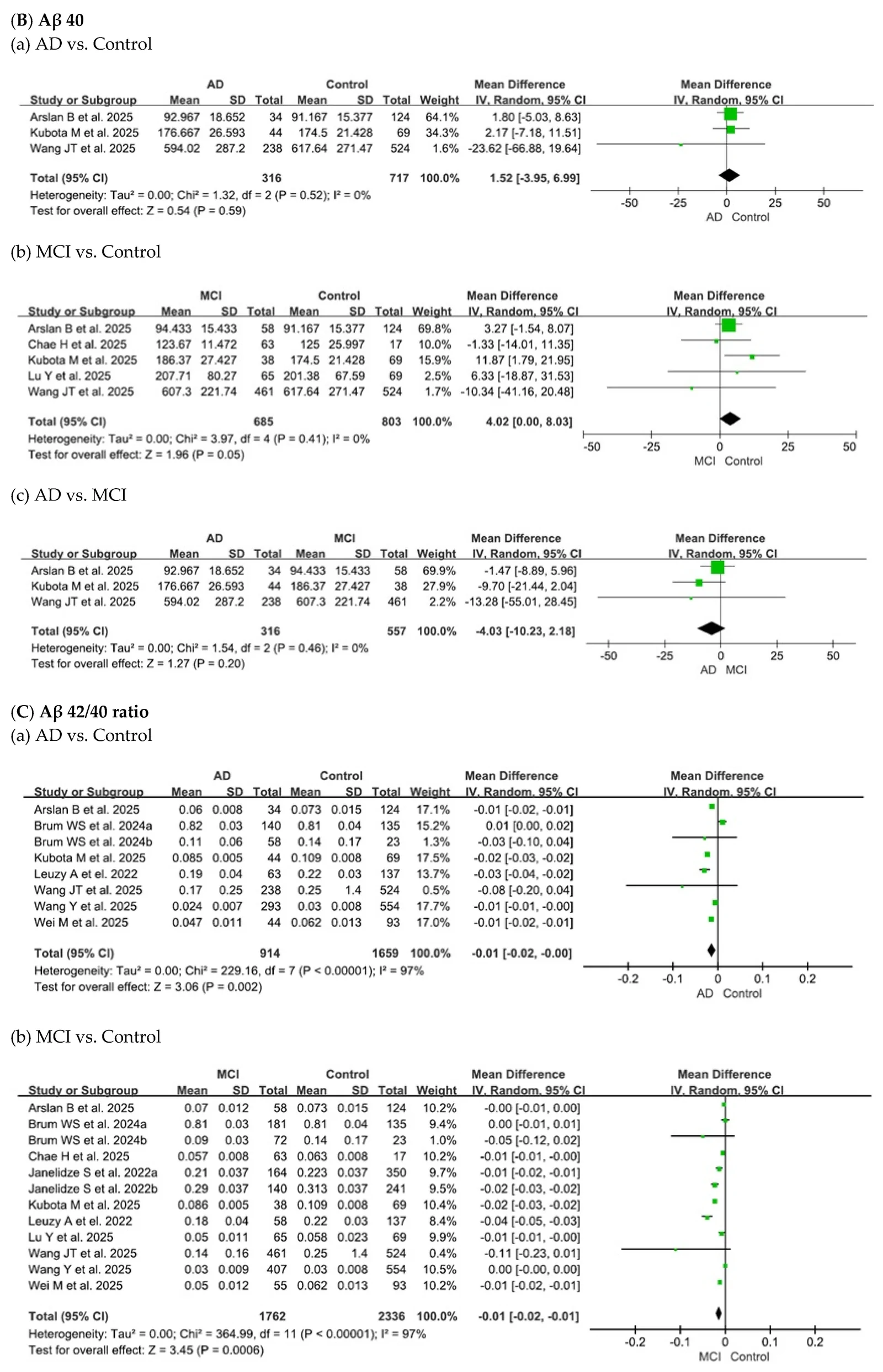

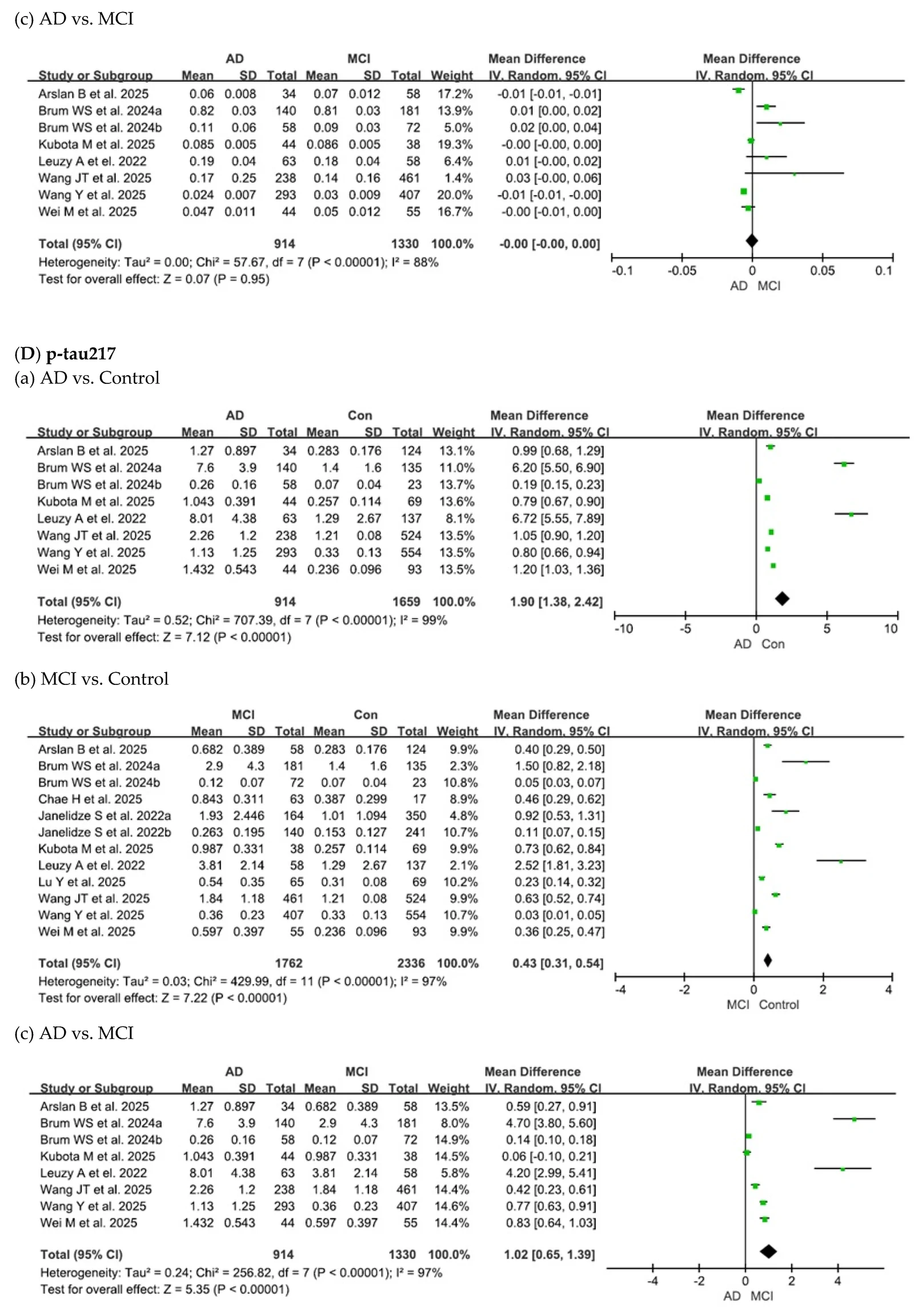

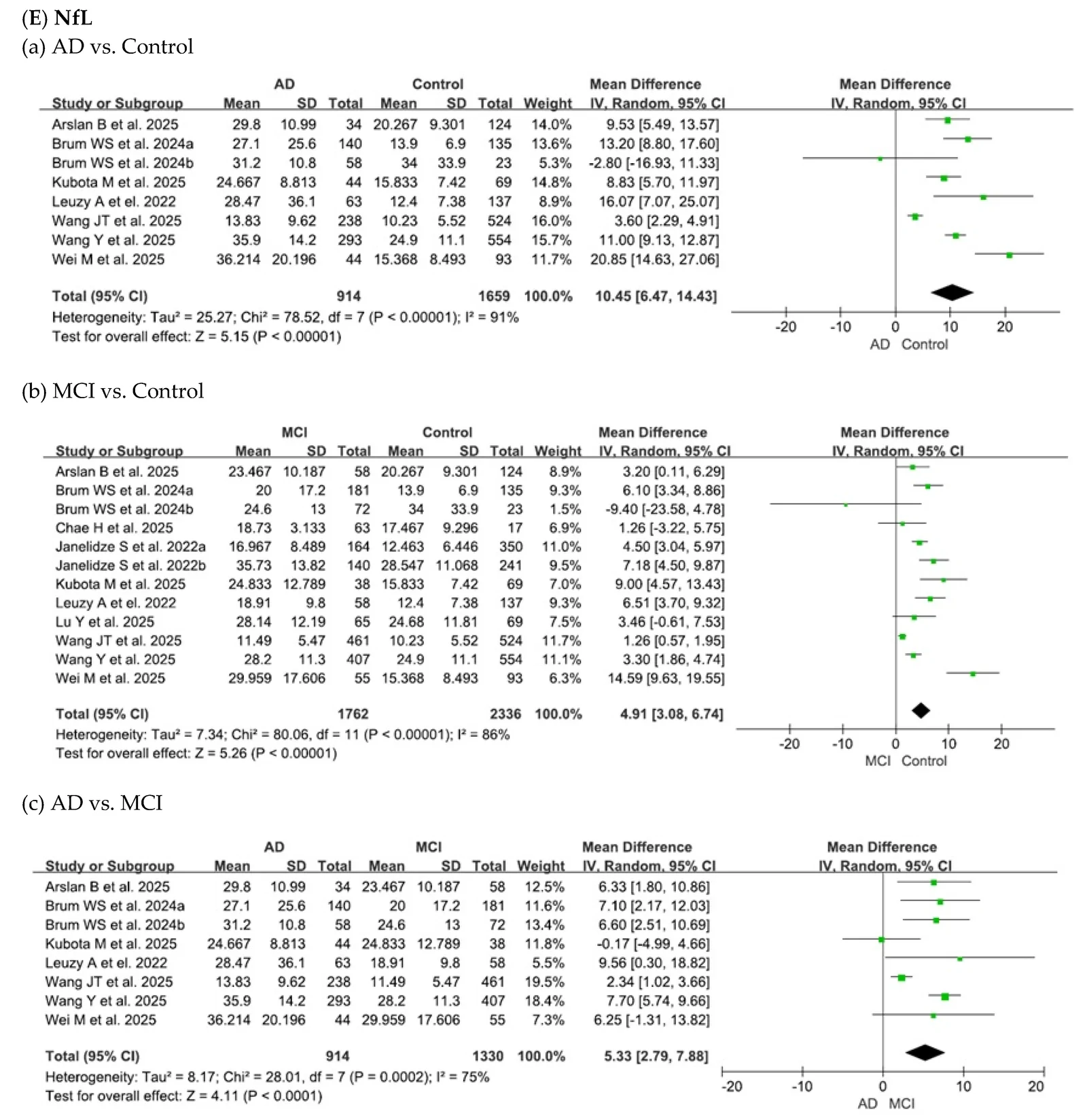
Forest plots of meta-analysis of blood biomarkers (Aβ42, Aβ40, Aβ42/40 ratio, p-tau217, and NfL) [16,17,19,20,22,25,27,28,30,31].

**Table 1:** General characteristics of studies.

Study	Country	Group	M/F	Age	Cognitive Assessment	Score	Tracer	Aβ-PET (+) (Criteria; %)	Biomarkers
Arslan B et al. 2025 [27]	Canada	Con	35/89	70.3 (65.7–74.7)	MMSE	29.0 (28.0–30.0)	[18F]AZD4694	SUVR > 1.55; 24.2%	Simoa: Aβ42, Aβ40, GFAP, NfL, pTau181. pTau217, pTau231, NTA-tau
		MCI	23/35	72.2 (66.3–75.9)		29.0 (27.0–29.0)		63.8%	
		AD	13/21	66.9 (61.1–73.5)		23.0 (20.0–25.8)		97.1%	
Brum WS et al. 2024 [22]	Canada (TRIAD)	Con	70/65	66.6 (8.9)	MMSE	28.7 (1.4)			Simoa: Aβ42, Aβ40, GFAP, NfL, pTau181. pTau217, pTau231
		MCI	101/80	70.9 (8.7)		27.0 (1.9)			
		AD	68/72	73.8 (7.0)		20.3 (4.3)			
	Sweden (BioFINDER-2)	Con	7/16	73.6 (5.8)	MMSE	25.2 (5.9)	[18F]AZD4694	26.1%	
		MCI	34/38	72.3 (5.8)		29.3 (0.89)		68.1%	
		AD	27/31	67.0 (8.3)		28.0 (1.9)		87.9%	
Chae H et al. 2025 [16]	South Korea	Con	7/10	70.8 (6.4)	K-MMSE	28		29.4%	Simoa: Aβ42, Aβ40, GFAP, NfL, pTau181. pTau217
		MCI	23/40	72.5 (6.3)		25		63.5%	
Fang L et al. 2025 [23]	China (Shenzhen, GHABS + STAR cohorts)	Con	50/107	62.6 (9.0)	MoCA	26.6 (2.2)	GHABS only, *n* = 107 PET (of 210): [18F]Florbetapir, [18F]D3FSP	β value: 0.784	Simoa: Aβ42, Aβ40, GFAP, NfL, pTau217
		MCI + AD	53	68.9 (9.3)		16.2 (6.2)		β value: 0.293	
Janelidze S et al. 2022 [28]	Sweden (BioFINDER-2)	Con	167/183	64 (53–75)	MMSE	29 (28–30)	[18F]Flutemetamol	SUVR 0.62 (0.59–0.66); 75 (22.0%)	Mass spectrometry: Aβ42, Aβ40, Simoa: NfL, Mesoscale Discovery platform: pTau217
		MCI	85/79	71 (66–76)		27 (25–29)		SUVR 0.72 (0.61–0.72); 83 (52.9%)	
	Sweden (BioFINDER-1)	Con	107/134	72 (68–75)	MMSE	29 (28–30)	[18F]Flutemetamol	SUVR 0.67 (0.64–0.77); 29.1%	
		MCI	90/50	71 (67–76)		27 (26–29)		SUVR 0.93 (0.70–1.09); 66.2%	
Jang H et al. 2025 [24]	South Korea (K-ROAD, 25 hospitals)	Con	238/428	71 (64–76)	MMSE	28 (27–29)	[18F]Florbetaben or [18F]flutemetamol	CL 5 (−1.5–22.5)	Simoa: Aβ42, Aβ40, GFAP, NfL, pTau217
		AD	2032	73 (66–78)		24 (20–27)		CL 67.51 (4.8–110.2)	
Kang SH et al. 2025 [29]	South Korea	Con	175/263	71.3 (7.3)	MMSE		[18F]Florbetaben or [18F]Flutemetamol		Simoa: Aβ42, Aβ40, GFAP, NfL, pTau217
		MCI	311/451	71.8 (8.0)					
		AD	178/330	70.3 (9.6)					
Kubota M et al. 2025 [30]	Japan	Con	35/34	69 (63–76)	MMSE	29.0 (29.0–30.0)	[18F]Florbetaben	CL −2.3 (−5.7–5.1)	Simoa: GFAP, NfL pTau181, pTau217 HISCL^®^: Aβ42, Aβ40
		MCI	21/17	76 (70–79)		27.0 (26.0–29.0)		CL 83.6 (58.3–105.0)	
		AD	20/24	75 (62–80)		21.0 (17.0–22.0)		CL 94.3 (64.3–118.2)	
Leuzy AL et al. 2022 [25]	Sweden (BioFINDER-2)	Con	73/64	72.57 (7.33)	MMSE	28.98 (1.21)	[18F]Flutemetamol	CL ≥19; 0%	Simoa: Aβ42/40, p-tau217, NfL
		MCI	26/32	71.79 (7.97)		26.69 (1.91)		0%	
		AD	28/35	73.06 (6.90)		19.74 (4.23)		100	
Lu Y et al. 2025 [17]	China	Con	69	63.93 (6.21)	MoCA-B/ACEIII	25.57 (2.31)/82.76 (6.38)	[18F]Florbetapir	Visual read; 0%	Simoa: Aβ42, Aβ40, Aβ42/40, t-tau, pTau181, pTau217, NfL, GFAP
		MCI	65	64.11 (5.91)		21.81 (4.08)/74.00 (9.67)		100%	
Pereira JB et al. 2021 [18]	Sweden (BioFINDER-2)	Con	52	69.2 (42.4–87.5)	MMSE	28.0 (23–30)	[18F]Flutemetamol	SUVR 0.79 (0.53–1.31); 22.6%	EUROIMMUN: Aβ42/40, Lilly: P-tau181, pTau217, Simoa: NfL
		MCI	67					33.3%	
		AD	156/67	73.9 (52.8–87.6)		20.1 (8–28)		100%	
Wang JT et al. 2025 [31]	China	Con	268/256	73.51 (6.49)	MoCA	24.40 (3.63)	[18F]Florbetapir	Visual read; 31.9%	SMID: Aβ42, Aβ40, Aβ42/40, GFAP, NfL, pTau181, pTau217
		MCI	179/282	74.62 (6.73)		14.77 (5.62)		100%	
		AD	74/164	74.20 (9.55)		8.61 (5.39)		100%	
Wang Y et al. 2025 [19]	China	Con	206/348	65.2 (7.3)	MoCA-BC/ACEIII-CV	25.5 (2.6)/91.8 (7.7)	[18F]Florbetapir	Visual read; 10%	LiCA/Simoa: Aβ42, Aβ40, GFAP, NfL, pTau181, pTau217
		MCI	163/244	68.1 (7.0)		21.1 (3.8)/70.6 (9.3)		28%	
		AD	119/174	69.2 (8.3)		12.7 (4.3)/49.0 (13.4)		75%	
Wei M et al. 2025 [20]	China	Con	34/59	65.118 (3.975)	MMSE/MoCA-B	28.956 (1.080)/25.962 (3.131)	[18F]Florbetapir	SUVR > 1.18; NR	Simoa: Aβ42/40, pTau181, pTau217, NfL, GFAP
		MCI	25/30	71.582 (6.688)		24.981 (3.406)/20.500 (3.580)		NR	
		AD	16/28	72.818 (7.289)		16.195 (6.165)/11.593 (4.693)		NR	

Note: Data are expressed as the mean ± standard deviation (SD) or the median [interquartile range]. The biomarkers column lists the plasma biomarkers measured in each study, preceded by the analytical platform used for quantification, where reported. In cells reporting amyloid-PET tracer/quantification method, “NR” indicates that this information was not specified in the source publication. Abbreviations: Aβ: amyloid beta; AD: Alzheimer’s disease; β value: standardized beta (β) values; CL: Centiloid; Con: cognitively unimpaired individuals; EUROIMMUN: EUROIMMUN immunoassays for plasma Aβ 42 and Aβ 40; GFAP: glial fibrillary acidic protein; LiCA: light-initiated chemiluminescent assays; Lilly: immunoassays developed at Lilly Research Laboratories; MCI: mild cognitive impairment; MMSE, Mini-Mental State Examination; MoCA-BC: Chinese version of Montreal Cognitive Assessment-Basic, NfL: neurofilament light chain; NR: not reported; NTA-tau: N-terminal containing tau fragments; pTau: phosphorylated tau; Simoa: single molecule array; SMID: Single Molecular Immunity Detection; SUVR: standardized uptake value ratio; TRIAD: Translational Biomarkers in Aging and Dementia (TRIAD) cohort; t-tau: total tau.

## Data Availability

The data supporting the findings of this study are available from the corresponding author upon reasonable request.

## Supplementary Materials

The following supporting information can be downloaded at: https://www.mdpi.com/article/10.3390/ijms27198574/s1.

## Abbreviations

The following abbreviations are used in this manuscript:

Aβ	Amyloid beta
ACE-III-CV	Chinese version of Addenbrooke’s Cognitive Examination III
AD	Alzheimer’s disease
ADAS	Alzheimer’s Disease Assessment Scale
ADNI	Alzheimer’s Disease Neuroimaging Initiative database
β value	Standardized beta (β) values
CANDI	China Aging and Neurodegenerative Disorder Initiative cohort
CDR-SB	Clinical Dementia Rating-Sum of Boxes
CI	Confidence interval
CL	Centiloid
Con	Cognitively unimpaired individuals
CSF	Cerebrospinal fluid
CU	Cognitively unimpaired
CU-	Aβ-negative cognitively unimpaired
CU+	Aβ-positive cognitively unimpaired
FDA	Food and Drug Administration
GFAP	Glial fibrillary acidic protein
IQR	Interquartile range
MCI	Mild cognitive impairment
MCI+	Aβ-positive mild cognitive impairment
MCSA	Mayo clinic study of aging
MMSE	Mini-Mental State Examination
MoCA-B	Chinese version of Montreal Cognitive Assessment-Basic
NfL	Neurofilament light chain
NR	Not reported
NTA-tau	N-terminal containing tau fragments
PET	Positron emission tomography
p-tau	Phosphorylated tau
RoBANS	Risk of Bias Assessment Tool for Non-randomized Studies
ROI	Region of interest
SD	Standard deviation
SUVR	Standard uptake value ratio
TRIAD	Translational Biomarkers in Aging and Dementia cohort
t-tau	Total tau
UCSF	University of California San Francisco cohort
