# Supplementary material for: Plasma Neurofilament Light Chain as a Complementary Biomarker for Alzheimer’s Disease and Mild Cognitive Impairment: A Systematic Review and Meta-Analysis
Source: Int J Mol Sci. 2026 Sep 25;27(19):8574. doi: 10.3390/ijms27198574 (PMC13664730; doi:10.3390/ijms27198574)
Supplement: Supplementary file 1 [file ijms-27-08574-s001.zip › Supplementary Table S1.pdf]

Supplementary Table S1. Summary of heterogeneity statistics for pooled biomarker comparisons

Biomarker	Comparison	Tau^2	Chi^2	df	P-value	I^2 (%)
Aβ42	AD vs Control	3.86	21.42	2	<0.0001	91
	MCI vs Control	1.33	20.20	4	0.0005	80
	AD vs MCI	0.00	0.37	2	0.83	0
Aβ40	AD vs Control	0.00	1.32	2	0.52	0
	MCI vs Control	0.00	3.97	4	0.41	0
	AD vs MCI	0.00	1.54	2	0.46	0
Aβ42/40 ratio	AD vs Control	0.00	229.16	7	<0.00001	97
	MCI vs Control	0.00	364.99	11	<0.00001	97
	AD vs MCI	0.00	57.67	7	<0.00001	88
p-tau217	AD vs Control	0.52	707.39	7	<0.00001	99
	MCI vs Control	0.03	429.99	11	<0.00001	97
	AD vs MCI	0.24	256.82	7	<0.00001	97
NfL	AD vs Control	25.27	78.52	7	<0.00001	91
	MCI vs Control	7.34	80.06	11	<0.00001	86
	AD vs MCI	8.17	28.01	7	0.0002	75

Note: Tau^2, between-study variance; Chi^2, Cochran's Q statistic; df, degrees of freedom; I^2, percentage of total variation across studies attributable to heterogeneity rather than chance. All pooled estimates were calculated using a random-effects (inverse variance) model in Review Manager 5.4 (RevMan 5; Cochrane Collaboration). AD, Alzheimer's disease; MCI, mild cognitive impairment; NfL, neurofilament light chain.
